# Sisters in structure but different in character, some benzaldehyde and cinnamaldehyde derivatives differentially tune *Aspergillus flavus* secondary metabolism

**DOI:** 10.1038/s41598-020-74574-z

**Published:** 2020-10-19

**Authors:** Franco Bisceglie, Francesca Degola, Dominga Rogolino, Gianluigi Giannelli, Nicolò Orsoni, Giorgio Spadola, Marianna Pioli, Francesco M. Restivo, Mauro Carcelli, Giorgio Pelosi

**Affiliations:** Department of Chemistry, Life Sciences and Environmental Sustainability, University of Parma, Parco Area delle Scienze 11/A, 43124 Parma, Italy

**Keywords:** Microbiology, Plant sciences, Environmental sciences

## Abstract

Great are the expectations for a new generation of antimicrobials, and strenuous are the research efforts towards the exploration of diverse molecular scaffolds—possibly of natural origin – aimed at the synthesis of new compounds against the spread of hazardous fungi. Also high but winding are the paths leading to the definition of biological targets specifically fitting the drug’s structural characteristics. The present study is addressed to inspect differential biological behaviours of cinnamaldehyde and benzaldehyde thiosemicarbazone scaffolds, exploiting the secondary metabolism of the mycotoxigenic phytopathogen *Aspergillus flavus*. Interestingly, owing to modifications on the parent chemical scaffold, some thiosemicarbazones displayed an increased specificity against one or more developmental processes (conidia germination, aflatoxin biosynthesis, sclerotia production) of *A. flavus* biology. Through the comparative analysis of results, the ligand-based screening strategy here described has allowed us to delineate which modifications are more promising for distinct purposes: from the control of mycotoxins contamination in food and feed commodities, to the environmental management of microbial pathogens, to the investigation of specific structure–activity features for new generation drug discovery.

## Introduction

All around the world, the spread of some phytopathogenic fungi on agricultural crops entails the contamination of derived products by mycotoxins, secondary metabolites produced by various fungal species, mainly belonging to *Aspergillus*, *Penicillium* and *Fusarium* genera^1^. Mycotoxin contamination of food and feed commodities represents a major threat from both an economic and a sanitary perspective, due to the high toxicity and carcinogenicity of such compounds to humans and animals. Amongst the mycotoxigenic species, *A. flavus* is one of the main producer of aflatoxins (AFs), and in particular of aflatoxin B, which is classified as the most cancerogenic natural compound and which has been proven to increase the risks for hepatocellular carcinoma in exposed individuals^2^. Dietary intake is the primary non-occupational source of human exposure to AFs, which have been found in a variety of agricultural commodities, but mostly in maize, peanuts, cottonseed, and tree nuts^3^. The risk of contamination is not limited to the field (pre-harvest) stage, since a carry-over phenomenon along the food chain can occur, and AFs might be retained during storage and/or processed by intermediate consumer (such as livestock), giving rise to metabolically modified forms that still pose a sanitary concern to the final consumer^4^. As *A. flavus* colonization on kernels represents the principal source of AF contamination of food and feed, many strategies have been developed to avoid, or at least to limit, the presence of the fungus on crops. Among them, the use of fungicides, the application of pesticides against insects that favour fungal infection or bio-competitive approaches that directly contain the growth/diffusion of aflatoxigenic strains are widely applied^5–9^. However, more recently, based on the need for more sustainable and “green” policies, the efforts of many scientists were devoted to unravel the regulatory mechanisms that control toxin accumulation, in order to design molecules that could more specifically target the mycotoxin biosynthetic apparatus.

AF biosynthesis in *A. flavus* depends on the coordinate expression of a set of genes clustered on chromosome III^10^, most of them encoding for enzymes directly involved in the multistep biosynthetic pathway, but at least two genes are known to encode for expression regulatory proteins. Nonetheless, other global regulators (including those controlling different metabolic pathways or developmental processes), encoded by genes located outside the aflatoxin cluster, were found to intervene in the control of the toxin synthesis^11–17^. It thus emerges that a complex network of enzymes and regulatory proteins is responsible for AF accumulation and each of them might be the candidate target for new potential inhibitors, for which an increased selectivity and specificity are also required to counteract the spread of resistant fungal strains.

To date, the methods to inhibit the AFs production essentially rely on the alteration of the physiological environment and/or the fungus *sensing*, for example via the perturbation of the oxidative stress-related enzymes^18^, on the modulation of signal transduction upstream the AFs gene cluster expression, as it has been demonstrated for the regulation factor of primary metabolism *VeA* and for the Ca^2+^ ion^19,20^, or on the blockage of specific enzymatic steps along the toxin biosynthetic pathway. Several terpenoids and other plant-derived natural compounds (such as cumarin, carotenoids, lutein, caffeine, limonene) were reported to be effective in preventing the final accumulation of AFs through the inhibition of enzymes directly involved in the conversion of precursors in AFs intermediates^6,21–23^. On the other hand, a variety of selective inhibitors of AFs has been found by screening libraries of natural or synthetic molecules^24,25^. This experimental approach allows, beside the discovery of compounds suitable for fighting contamination of agricultural commodities, to acquire new insights into the regulatory mechanisms governing the toxin metabolism. This mostly represents a noteworthy advantage for those strategies that keep an eye on a wider philosophy of environmental intervention, trying to avoid or reduce side-effects on other microbial species that share the same ecological niche with the relevant mycotoxigenic fungi.

**Figure 1:**
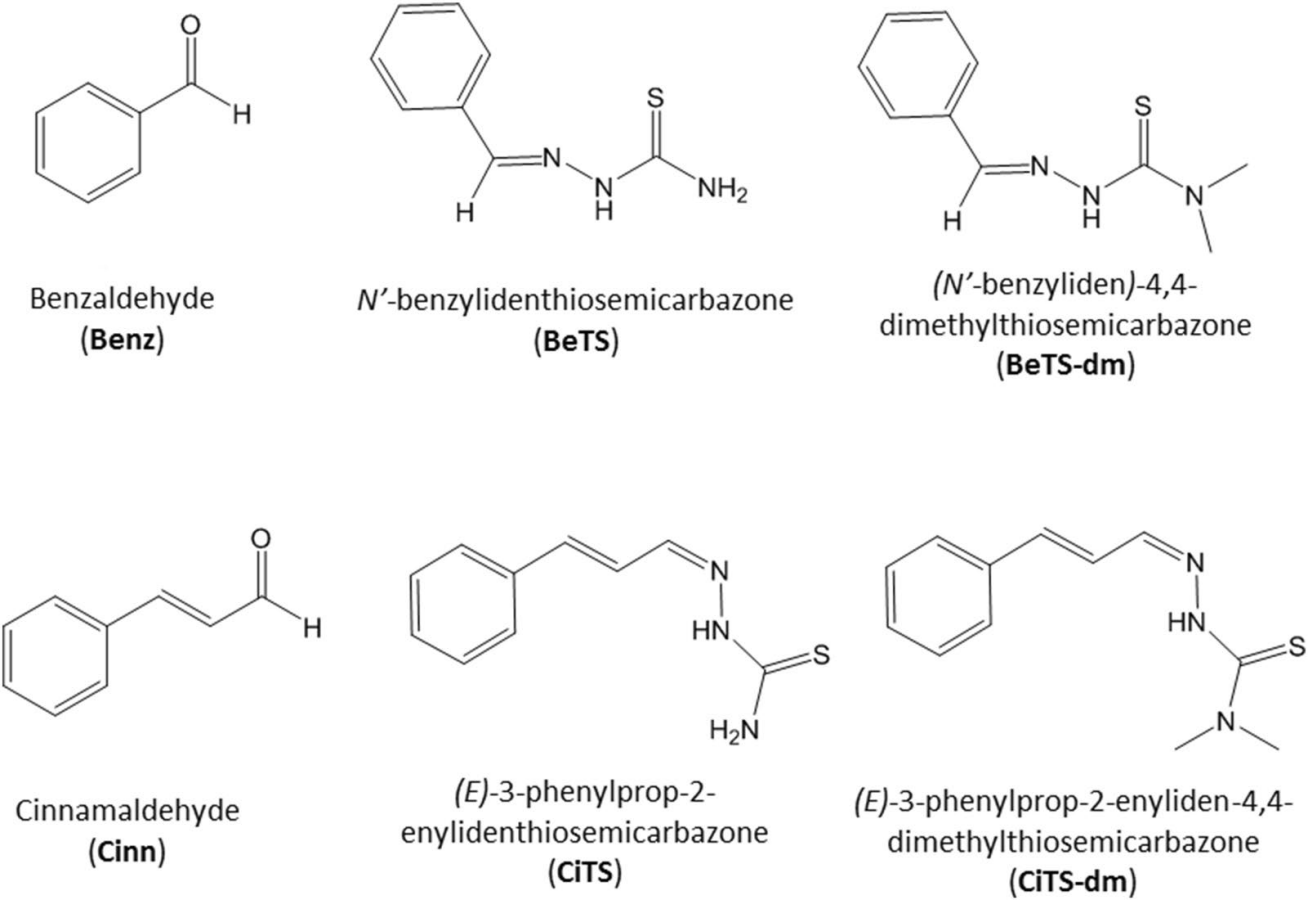
Schematic representation of the structures of benzaldehyde (Benz), cinnamaldehyde (Cinn), their thiosemicarbazone derivatives BeTS, and CiTS, and their di-methylated variants BeTS-dm, and CiTS-dm.

We recently reported several sets of data concerning the biological activity of molecules, belonging to the class of thiosemicarbazones (TS), that displayed different specificity against one or more developmental regulated process (conidia germination, toxin biosynthesis, sclerotia production) in *A. flavus*^26–30^: here we compare and investigate how, owing to scaffold modifications, TS derivatives of benzaldehyde and cinnamaldehyde (Fig. 1) change in their antifungal and anti-toxigenic effect, unravelling which structural characteristic is responsible for the observed, specific biological activities.

## Materials and methods

### Chemistry

Chemicals for synthesis were purchased from Sigma-Aldrich Srl (Milano, Italy) and used without further purification. The purity of the compounds was determined by elemental analysis and verified to be ≥ 95%. ^1^H-NMR spectra were obtained in a 5 mm NMR precision tube at 298 K on a Bruker Avance 400 FT spectrophotometer. Elemental analyses were performed by using a FlashSmartCHNS analyser (Thermo Fisher) with gas-chromatographic separation. The MS spectra were recorded in methanol and acquired in positive EI mode by means of a DEP-probe (Direct Exposure Probe) mounting on the tip of a Re-filament with a DSQII Thermo Fisher apparatus, equipped with a single quadrupole analyser or performing an ESI–MS analysis using a Waters Acquity Ultraperformance ESI–MS spectrometer with Single Quadrupole Detector.

The synthesis of the TSs (Fig. 1) was accomplished following a protocol we have previously reported^29,31^. Briefly, the aldehyde was dissolved in hot ethanol and added of few drops of glacial acetic acid. An equimolar amount of the proper thiosemicarbazide was added to the solution and the reaction was heated under reflux for 4–6 h. The solution was cooled r.t. and the TSs were isolated as solids by filtration, washed several times with cold ethanol and ether and then dried under vacuum.

#### *(E)-N’-benzylidenthiosemicarbazone* (**BeTS**).

Pale yellow solid. Yield: 45%. M.p. = 154–155 °C. ^1^H-NMR (DMSO-d_6_, 25 °C), δ: 11.43 (s, 1H, NH); 8.20 (s, 1H, CH=N); 8.06 (s, 1H, NH); 7.99 (s, 1H, NH); 7.80 (br, 2H, CH_Ar_); 7.40 (m, 3H, CH_Ar_). ^13^C NMR (DMSO-d_6_, 25 °C), δ: 178.5 (C=S), 142.8 (C=N), 134.8, 130.5, 128.9, 127.9 (Ph). EI-MS (positive ions, CH_3_OH): m/z = 179.1 [M]^+^. Anal. Calcd. for C_8_H_9_N_3_S: C, 53.61; H, 5.06; N, 23.44; S, 17.89. Found: C 53.58; H 5.14; N 23.36; S 17.94.

#### *(E)- N’-benzyliden-4,4-dimethylthiosemicarbazone* (**BeTS-dm**).

Yellow solid. Yield: 57%. M.p. = 159–160 °C. ^1^H-NMR (DMSO-d_6_, 25 °C), δ: 10.95 (s, 1H, NNH); 8.20 (s, 1H, CH=N); 7.64 (d, 2H, J = 7 Hz, CH_Ar_); 7.38 (m, 3H, CH_Ar_); 3.30 (s, 6H, NCH_3_). ^13^C NMR (CDCl_3_, 25 °C), δ: 181.8 (C=S), 142.9 (C=N), 134.1, 130.5, 129.2, 127.5 (Ph), 44.5(CH_3_). EI-MS (positive ions, CH_3_OH): m/z = 207.1 [M]^+^. Anal. Calcd. for C_10_H_13_N_3_S: C 57.94; H 6.32; N 20.27; S 15.47. Found: C 57.82; H 6.51; N 20.23; S 15.52.

#### *(E)-3-phenylprop-2-enylidenthiosemicarbazone* (**CiTS**).

White powder. Yield: 96%. M.p. = 139 °C. ^1^H-NMR (DMSO-d_6_, 25 °C) δ: 11.40 (s, 1H, NNH); 8.18 (s, 1H, NH); 7.92 (d, J = 9.2 Hz, 1H, CH=C); 7.61 (s, 1H, NH); 7.58 (d, J = 8.2 Hz, 2H, CH_Ar_); 7.39 (t, J = 8.2 Hz, 1H, CH_Ar_); 7.34 (t, J = 8.2 Hz, 2H, CH_Ar_); 7.04 (d, J = 16.1 Hz, 1H, CH=N); 6.87(dd, J = 16.1, J′ = 9.2 Hz, 1H, CH=C). ^13^C-NMR (DMSO-D_6_, 25 °C), δ: 178.09 (C=S), 145.22 (C=N), 139.36 (C=C), 136.31 (C=C), 129.36 (C–H aromatic), 127.40 (C–H aromatic), 125.51 (C ipso). ESI–MS (positive ions, CH_3_OH): m/z = 206.09 [M]^+^. Anal. calc. for C_10_H_11_N_3_S: C 58.51, H 5.40, N 20.47, S 15.62. Found: C 58.73, H 5.44, N 20.52, S 15.73%.

#### *(E)-3-phenylprop-2-enyliden-4,4-dimethylthiosemicarbazone* (**CiTS-dm**).

Orange powder. Yield: 79%. M.p. = 140 °C. ^1^H-NMR (DMSO-d_6_, 25 °C), δ: 10.83 (s, 1H, NH); 8.04 (dd, J = 6.1 Hz, J′ = 2.5 Hz, 1H, CH=C); 7. 61 (d, J = 7.2 Hz, 2H, CH=C + CH=N); 7.40 (t, J = 7.2 Hz, 2H, CH_Ar_); 7.33 (t, J = 7.2 Hz, 1H, CH_Ar_); 6.97 (m, 2H, CH_Ar_); 3.26 (s, 6H, 2CH_3_). ^13^C-NMR (DMSO-D_6_, 25 °C), δ: 180.52 (C=S), 147.13 (C=N), 128.38 (C=C), 136.49 (C=C), 129.30 (C–H aromatic), 127.41 (C–H aromatic), 126.06 (C ipso), 42.29 (CH_3_). ESI–MS (positive ions, CH_3_OH): m/z = 234.57 [M]^+^. Anal. calc. for C_12_H_15_N_3_S: C 61.77, H 6.48, N 18.01, S 13.74. Found: C 61.82, H 6.53, N 17.89, S 13.84%.

Compounds were finally dissolved in dimethyl sulfoxide (DMSO; CARLO ERBA Reagents Srl, Milano, Italy), to obtain 10 mM stocks.

### DPPH radical scavenging activity assay

Scavenging activity of thiosemicarbazones was assessed against the 2,2-diphenyl-1-picrylhydrazyl radical (DPPH⋅), in accordance with Choi et al.^32^. Molecules were tested at 5, 20, 30, 40 and 50 μM concentration: freshly prepared 90 μM DPPH⋅ dissolved in methanol (1 mL aliquot) was added to 4 mL solution containing the test compound. The resulting mixture was stirred and then incubated for 30 min at room temperature. Scavenging activity of TSs against DPPH⋅ was evaluated as the decrease in absorbance, measured at 518 nm, of the solution, in comparison with 0.3 mM ascorbic acid. Values were expressed as percentage inhibition of DPPH absorbance in relation to the control values without the thiosemicarbazone (ascorbic acid maximal inhibition was considered 100%).

### Fungal strains and culture conditions

Two wild strains of *A. flavus* (the aflatoxigenic and sclerotigen strain CR10, and the non-toxigenic strain TOφ) were used to assay the biological activity of TSs, as previously reported^30^, and are available on request from the corresponding author. Strains maintenance and conidia suspensions were obtained in YES-agar [2% (w/v) yeast extract (Difco, Detroit, MI), 5% (w/v) sucrose (Sigma, St Louis, MO), 2% (w/v) agar (Difco, Detroit, MI)], according to Degola et al.^33^. A coconut clarified medium (CCM) was used for AF determination^34^.

### Antifungal activity

Mycelium early development (post-germination hyphal growth) was assessed by inoculating 5 × 10^3^ of CR10 conidia in 96 multiwell plates (Sarstedt, Newton, NC, USA), in a final volume of 200 μL/well of YES liquid medium amended with test compounds at increasing concentrations (from 25 to 50 µM), and analysing changes in optical density after 46 h of static growth at 28 °C. DMSO (0.25, 0.5 and 1% v/v respectively) was used as control. The optical density was recorded at 620 nm for each well with a microplate reader (TECAN SpectraFluor Plus microplate reader, Männedorf, Switzerland) without shaking. Samples were inoculated in quadruplicate. Values were then converted to percentage inhibition with respect to the relevant control (DMSO-treated cultures), and expressed as means ± S.D.

Mycelium biomass production was assessed after six days of incubation: mycelia from single wells were recovered, slightly dried on paper towels, and weighed. Values were then converted to percentage inhibition with respect to the relevant control (DMSO-treated cultures), and expressed as means ± S.D.

### Interference with *A. flavus* secondary metabolism: AFs accumulation and sclerotia biogenesis assays

Aflatoxin production was assessed by the microplate fluorescence-based procedure described in Degola et al.^34^. Coconut-derived medium was added with compounds at increasing concentrations [25, 50 and 100 µM; 0.5% (v/v) DMSO was used as control]; CCM cultures were incubated at 25 °C in the dark, under stationary conditions for 6 days, and AFs accumulation was directly evaluated in the culture medium by fluorescence emission determination (TECAN SpectraFluor Plus microplate reader, Männedorf, Switzerland; λ_ex_ = 360 nm; λ_em_ = 465 nm; manual gain = 83; lag time = 0 µs; number of flashes = 3; integration time = 200 µs). Samples were inoculated in quadruplicate; experiments were conducted in triplicate. Values were then converted to percentage inhibition with respect to the relevant control (DMSO-treated cultures), and expressed as means ± S.D.

Sclerotia biogenesis was evaluated in Czapek medium [3% (w/v) sucrose, 0.3% (w/v) sodium nitrate, 0.1% (w/v) di-potassium hydrogen phosphate, 0.05% (w/v) potassium chloride, 0.05% (w/v) magnesium sulfate heptahydrate, 1.5% (w/v) agar]: CR10 strain was point-inoculated on agar plates amended with compounds at the final concentration of 50 µM, then, after 10 days of incubation at 30 °C, sclerotia were manually recovered by scratching the colonies surface, ethanol-washed, dried up at 60 °C and weighed. Plates were inoculated in four replicates. Values were then converted to percentage inhibition with respect to the relevant control (DMSO-treated cultures), and expressed as means ± S.D.

### *Saccharomyces cerevisiae* strain and asci production (gametogenesis) assay

The diploid W303 yeast strain (a/α ade2 leu2 ura3 trp1 his3) was used, according to Dallabona et al.^35^: cells from YP solid cultures [1% (w/v) yeast extract, 2% (w/v) peptone, 2% (w/v) glucose], incubated for three days at 28 °C, were recovered and straight-inoculated on the surface of SPO IV medium [0.25% (w/v) yeast extract, 2% (w/v) K-acetate, 0.1% (w/v) glucose] agar plates, supplemented with compounds at the final concentration of 50 µM; 0.5% (v/v) DMSO-amended cultures were used as negative control, while a treatment with 50 µM of the 2-isopropylbenzaldehyde TS (mHtcum) was added as a positive control. After 6 days at 28 °C, a small volume of cells was sampled from each treatment, resuspended in bidistilled water, loaded on a glass slide for the observation at 200x magnification with an inverted microscope. Three replicates from each treatment were evaluated, and a total of five fields from each sample was counted. Experiments were conducted in triplicate.

### Total RNA extraction and gene expression analysis

Total RNA was extracted from 200 mg of mycelium sampled from 96-h-old CCM cultures amended with BeTS-dm or CiTS-dm 50 µM (0.5% v/v DMSO-amended cultures were used as control), following TRIzol Kit (Sigma-Aldrich, Saint Louis, MO, USA) procedure. Reverse-transcription of 2 µg of total RNA was obtained with Maxima First-Strand cDNA Synthesis Kit for qRT-PCR with dsDNase (Thermo Fisher Scientific, Waltham, MA, USA), following the manufacturer instructions. The complementary DNA samples were used as templates of qPCR reactions conducted with ABI 7300 instrumentation (Thermo Fisher Scientific, Waltham, MA, USA) and iTaq Universal SYBR Green Supermix (BioRad, Hercules, CA, USA). Primers sequences are reported in Table S1; *tub1* gene was used as housekeeping, and the expression level of targets was normalized accordingly. The ΔCT was calculated as CT_target gene_—ΔCT_internal standard_; the expression level variations were then expressed as 2^−ΔΔCT^ (with ΔΔCT = CT_treatment_—CT_control_). Three biological and three technical replicates per condition were performed. Amplification was conducted as follows: 2 min 50 °C, 10 min 95 °C; 15 s 95 °C and 1 min 60 °C (40 × cycles); dissociation curve was obtained with 15 s 95 °C, 1 min 60 °C, 15 s 95 °C and 15 s 60 °C.

### *A. flavus* proteome analysis

#### Two-dimensional Electrophoresis

The analysis of differentially expressed proteins in *A. flavus* exposed to TSs (BeTS, CiTS and their di-methylated derivatives) was conducted by two dimensional electrophoresis (2D PAGE). As previously described^28^, mycelia from 96-h-CCM microplate cultures (200 mg each) was frozen in liquid nitrogen, ground into a powder and added with 200 μL of lysis buffer [50 mM Tris–HCl pH 7.5, 2 M thiourea, 7 M urea, 2% (v/v) Triton X-100, 1% di-thiothreitol (DTT), 2% (w/v) soluble polyvinylpolypyrrolidone (PVPP), 1 mM phenylmethylsulphonylfluoride (PMSF), and 0.2% (v/v) β-mercaptoethanol]. Samples were centrifuged twice for 20 min at 13,000 × g before adding 200 μL 45% (w/v) of trichloroacetic acid to the supernatant, then incubated on ice for 10 min and centrifuged again for 15 min at 13,000 × g at 4 °C; pellets were washed with cold acetone for three times, then dried under a vacuum pump and resuspended in rehydration buffer [8 M urea, 2% (w/v) 3-[(3-cholamidopropyl)dimethylammonium]-1-propanesulfonate hydrate (CHAPS)]. Total proteins were quantified according to Bradford^36^, with bovine serum albumin as standard.

A total of 125 μg of total proteins was loaded on 7 cm strips (BioRad, Hercules, CA, USA. pH 3–10 and 5–8 range) isoelectrofocused and separated in a 12% polyacrylamide gels. Gels were stained with SYPRO Ruby Protein Stain (BioRad, Hercules, CA, USA), then the 2-D gel image elaboration and analysis were carried out with the PDQuest software (version 8.0.1; BioRad, Hercules, CA, USA; https://www.bio-rad.com/it-it/product/pdquest-2-d-analysis-software?ID=966deb78-2656-437f-b7a4-ab0a9bd45c8d). Three technical replicates for each of the three biological replicates were performed, for a total of nine gels for each class (control and treated samples). The built-in BioRad software “total quantity in valid spots” was used for normalization of spot volumes. Spot volumes of the replicated gels were compared and analyzed according to Student’s *t*-test, inferring statistical significance at *p* ≤ 0.01. In addition, only spots whose volumes varied significantly by at least a ratio of two and displayed reproducible change patterns (in at least two of the biological replicates and two of the corresponding technical replicates) were considered for MS analysis. Spots found to be differentially expressed were manually recovered from the gels, subjected to an in-gel digestion, and addressed to a MALDI-TOF/TOF analysis.

#### In-gel digestion and MALDI-TOF/TOF analysis

Peptide analysis was performed at the Proteomics Technology Facility, Department of Biology, University of York (https://www.york.ac.uk/depts/biol/tf/proteomics), as described in Margaria and Palmano^37^. Briefly: once being washed two times with 50% v/v ACN and 25 mM ammonium bicarbonate, and one with ACN, gel spots were dried in a vacuum concentrator for 20 min. Samples were rehydrated with 10 µL of trypsin 0.02 µg/µL solution [Promega, Madison, WI, USA] (trypsin was dissolved in 50 mM acetic acid then diluted five-fold by adding 25 mM ammonium bicarbonate to the final concentration). After overnight incubation at 37 °C, 1 µL aliquot of each peptide mixture was applied directly to the ground steel MALDI target plate, with an equal volume of 5 mg/mL CHCA solution [Sigma, St. Louis, MO, USA] in 50% v/v ACN containing 0.1% v/v TFA. Positive-ion MALDI mass spectra were obtained using a Ultraflex III MALDI TOF/TOF mass spectrometer [Bruker, Billerica, MA, USA] in reflectron mode, equipped with a Nd:YAG smart beam laser as described in Fazeli et al.^38^. Mass spectra were acquired over a mass range of m/z 800–4000, then final MS spectra were externally calibrated against an adjacent spot containing six peptides [des-Arg1-Bradykinin, 904.681; Angiotensin I, 1296.685; Glu1-Fibrinopeptide B, 1750.677; ACTH (1–17 clip), 2093.086; ACTH (18–39 clip), 2465.198; ACTH (7–38 clip), 3657.929]. A SNAP algorithm (C 4.9384, N 1.3577, O 1.4773, S 0.0417, H 7.7583) was used to obtain monoisotopic masses, fixing an S/N threshold of 2. Fragmentation was performed in LIFT mode without the introduction of a collision gas. The default calibration was used for MS/MS spectra, which were baseline subtracted and smoothed (Savitsky-Golay, width 0.15 m/z, cycles 4); monoisotopic peak detection used a SNAP averagine algorithm (C 4.9384, N 1.3577, O 1.4773, S 0.0417, H 7.7583) with a minimum S/N of 6. Peak list generation and spectral processing were obtained by using the Flex Analysis software version 3.3 (Bruker; https://www.bruker.com/service/support-upgrades/software-downloads/mass-spectrometry.html).

#### Database search and proteins identification

Tandem mass spectral data were submitted to database searching using the MASCOT program (Matrix Science, version 2.6.1), through the Bruker ProteinScape interface (version 2.1). Searched database was the Aspergillus subset of UniProt database (search criteria: Enzyme, Trypsin; Fixed modifications, Carbamidomethyl; Variable modifications, Oxidation; Peptide tolerance, 250 ppm; MS/MS tolerance, 0.5 Da; Max missed cleavages: 1. Significance threshold: *p* ≤ 0.05). Limits for minimal sequence coverage and difference between hypothetical and experimental mass were fixed at 10 and 25%, respectively. The theoretical mass and pI of the identified proteins were calculated from sequence data with the Expasy Compute pI/Mw tool.

### Statistical analysis

For statistical analyses, one-way analysis of variance (ANOVA) was used in the Past 3.x software (https://past.en.lo4d.com/windows)^39^. Results of scavenging radical potential, mycelium early growth, biomass production, AF accumulation, sclerotia biogenesis and asci production were analysed by Tukey’s test; differences were considered significant at *p* ≤ 0.01. Significance of relative expression ratios in gene expression analysis was determined via Mann–Whitney test (*p* ≤ 0.05).

## Results

### Chemistry

Thiosemicarbazones BeTS, BeTS-dm, CiTS and CiTS-dm (Fig. 1) were obtained in high yields by reacting benzaldehyde or cinnamaldehyde and thiosemicarbazide or 4,4-dimethyl-3-thiosemicarbazide^29,31^. The modification at the NH_2_ terminal moiety was designed to highlight how a modulation in lipophilicity and hydrogen-bonding capabilities could impact activity. Spectroscopic data relative to the characterization of the TSs are reported in the Experimental Section. For this class of ligands, literature data report a possible *E/Z* isomerization around the C=N double bond. In our case, the ^1^H-NMR spectra of all TSs in d_6_-DMSO evidenced only one set of signals, that can be related to the *E* isomer. The NH proton is observed at about 11 ppm, as expected. The ^13^C NMR is in agreement with the formation of the proposed thiosemicarbazone. Mass spectroscopy and elemental analysis confirmed the nature of the ligands.

### Evaluation of antioxidant activity by DPPH⋅ radical scavenging

**Figure 2:**
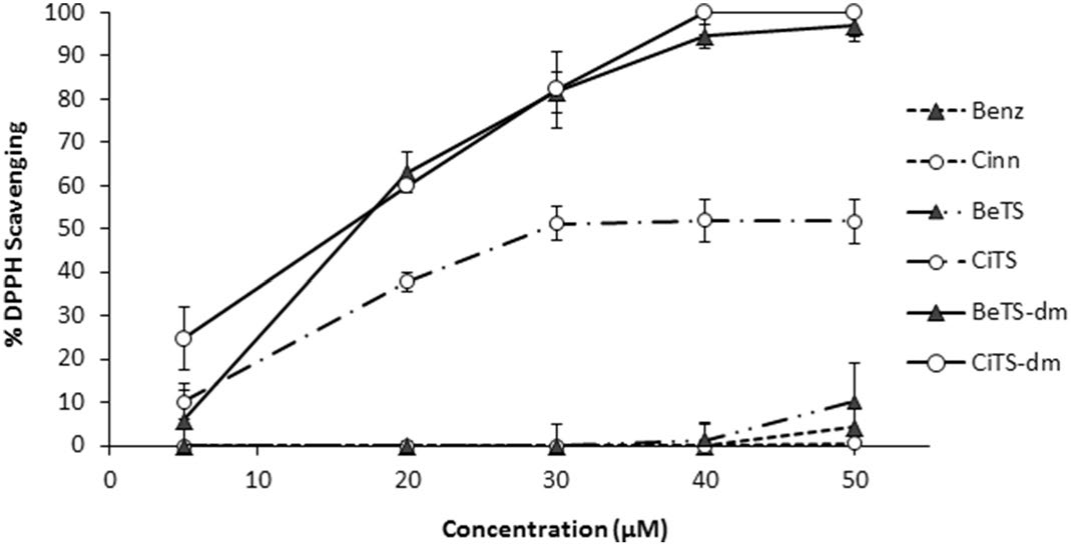
In vitro anti-oxidant assay (DPPH assay). The scavenging activity of compounds was tested at increasing concentrations (from 5 to 50 μM), and expressed as inhibition percentage with respect to ascorbic acid (0.3 mM) scavenging activity (100%). Values were presented as mean ± S.D. (n = 3).

At first, since a huge amount of data has been collected demonstrating how important it is for the fungal cell to have a strict control of its redox balance in order to coordinate growth, metabolism and development^40,41^, the antioxidant activity of each compound was determined. As reported in Fig. 2, neither the parent benzaldehyde (Benz) nor cinnamaldehyde (Cinn) displayed a noticeable scavenging activity at any of the tested concentrations (from 5 to 50 µM), as compared to ascorbic acid, taken as a reference at 0.3 mM concentration. BeTS was also ineffective in ROS scavenging, whereas CiTS showed an increase of the antioxidant activity with respect to the corresponding aldehyde (Cinn), reaching its maximum effect at 30 µM (50% DPPH⋅ scavenging). The N^4^ dimethylation in BeTS-dm and CiTS-dm improved the antioxidant activity of the parent compounds (BeTS and CiTS), showing a dose-dependent scavenging activity that reached 100% at the 40 µM concentration. At the lowest dose (5 µM) CiTS was more effective than both aldehydes and BeTS at the highest concentration (50 µM).

### Early and late effect on *A. flavus* growth

**Figure 3:**
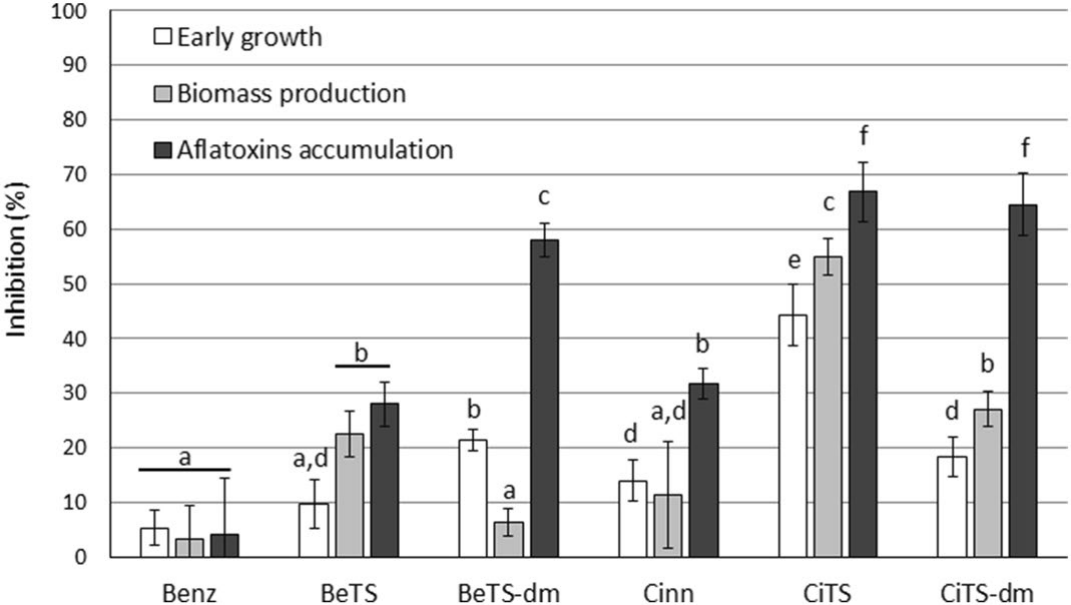
Biological activity of the tested compounds on *A. flavus*. The inhibitory activity of molecules on early development, biomass production and aflatoxin accumulation at 50 µM concentration was reported. Results were expressed as percentage with respect to control (0.5% DMSO-treated cultures). Different letters indicate statistically different values, at *p* value ≤ 0.01.

To evaluate the effect of the studied compounds on *A. flavus* growth, two assays were performed. The first procedure was addressed to detect the activity of the tested molecules during conidia germination (intended as hyphae extrusion) and the early phase of hyphae extension. The second assay, an end-point determination, measured the mycelium biomass production, that gives an idea of the cumulative effect of the relevant molecules on conidia germination and hyphae elongation/ramification during the mid-late mycelium growth. As shown in Figure S1-A, a slightly significant effect on germination was obtained by Benz, Cinn and BeTs at any tested concentration (≤ 20% of inhibition at 25, 50 and 100 µM); the same was observed for Benz and Cinn when compounds were tested for their effect on early fungal growth (Figure S1-B; Fig. 3). The TSs behaved differently: BeTS overcame the threshold of 20% inhibition at the highest concentration (30% inhibition at 100 µM), whereas CiTS showed a dose-dependent response that led the inhibition of fungal growth to range from 20 to 65% in terms of germination and from 45 to 70% in terms of biomass. Surprisingly, the dimethylated derivatives (BeTS-dm and CiTS-dm) were less effective than the unsubstituted ones BeTS and CiTS, even if a 40% inhibition of biomass accumulation was observed in 100 µM CiTS-dm treated cultures. Moreover, a different inhibition pattern was observed for the benzaldehyde derived compounds: BeTS was more effective on biomass accumulation than on fungal germination, while the contrary was assessed for BeTS-dm.

### Aflatoxin biosynthesis inhibition

To assess the effect of the studied compounds on AFs accumulation by *A. flavus*, a high throughput procedure previously described^28^ was used. Coconut-derived culture medium was supplemented with 25, 50 and 100 µM solution of molecules. As reported in Figure S1-C, Benz was ineffective in preventing the toxin accumulation (< 20% inhibition) at any concentration tested, while the effectiveness of its TS derivative BeTS was increased. If compared to Benz, Cinn has higher inhibitory activity, in particular at the highest dose (50% inhibition at 100 µM); as observed for BeTS, a higher, dose-dependent inhibition pattern was obtained for CiTS compared to its parent aldehyde. However, while the addition of a dimethyl group to the TS scaffold increased the anti-toxigenic activity of BeTS significantly (up to 80% inhibition for BeTS-dm at 100 µM), the dimethylation of N^4^ in CiTS (CiTS-dm) did not substantially modify the inhibitory effect on AF accumulation at any concentration tested (Figure S1-C; Fig. 3).

### Developmental analysis

**Figure 4:**
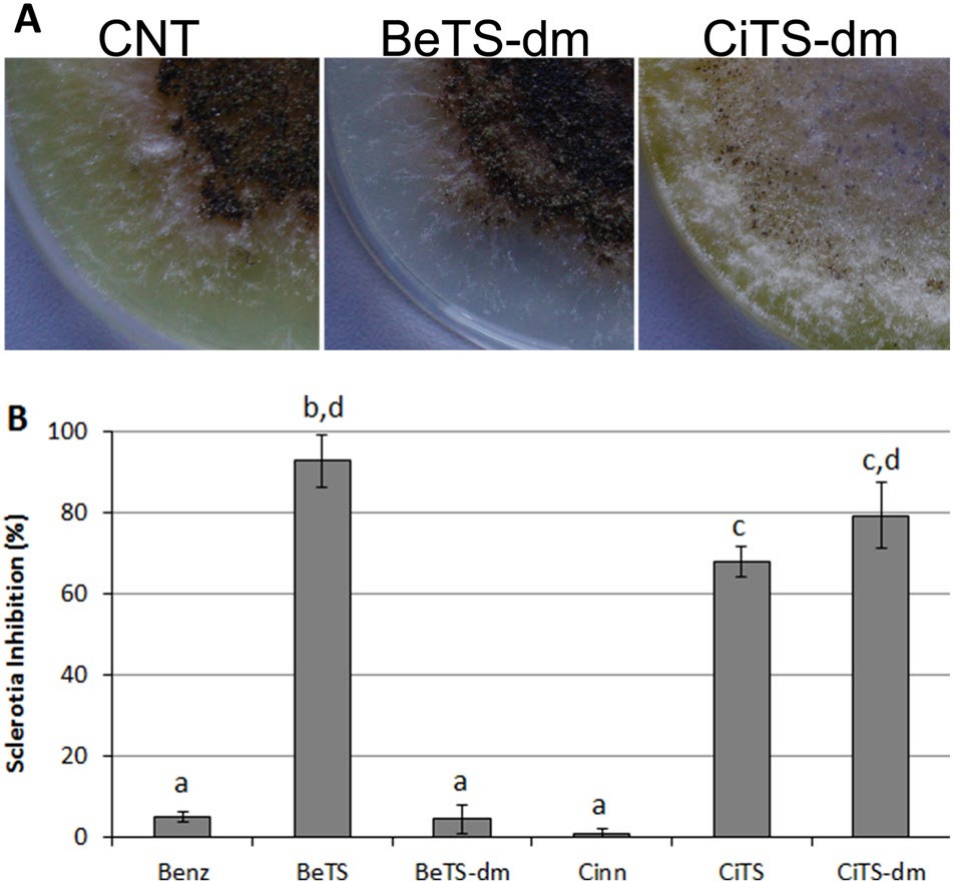
Effect of the tested compounds on sclerotia biogenesis. (**A**) The inhibitory activity of molecules on the sclerotia development in *A. flavus* cultures treated with 50 µM concentration. (**B**) Results were expressed as percentage with respect to 0.5% DMSO cultures (CNT). Different letters indicate statistically different values, at *p* value ≤ 0.01.

On the basis that in *A. flavus* many genetic links regulating secondary metabolites biosynthesis, and in particular AFs, also control the development of survival and sporogenically germinating, dispersal structures, we evaluated the effect of our molecules on sclerotia production. *A. flavus* Czapek solid cultures were amended with 50 µM of each compound; the biomass of the collected sclerotia was weighed and reported in Fig. 4B, expressed as percentage inhibition with respect to the control (0.5% DMSO cultures). Results showed a different behaviour amongst the two chemical families: both the aldehydes did not significantly vary from the control, while their derived TSs determined an abatement in sclerotia biomass of 70 and 90% for CiTS and BeTS, respectively. However, while the inhibitory effect recorded for the di-methylated Cinn derivative (analogous to that of CiTS) was somehow expected, due to the same trend previously observed for its anti-aflatoxigenic activity, totally bewildering was the finding that BeTS-dm interference on sclerotia development reverted at its aldehyde level (Fig. 4). This was apparently in contrast with the hypothesis of a cellular target, shared by the two dimethylated TSs, sufficiently upstream along the secondary metabolism to coordinately regulate both the AFs and the sclerotia production pathways.

### Gametogenesis impairment in *S. cerevisiae*

**Figure 5:**
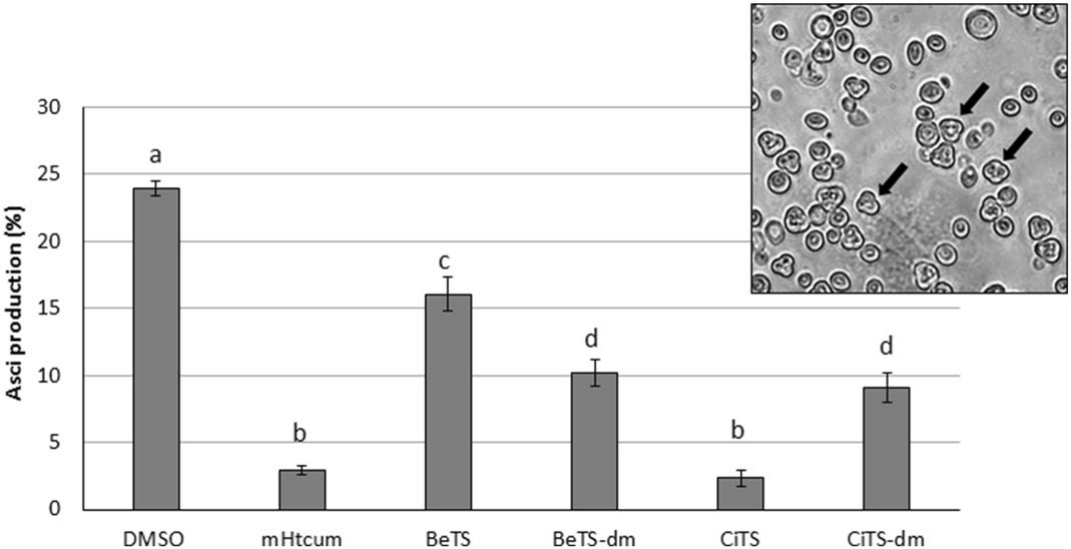
Asci production in W303 *S. cerevisiae* diploid strain treated with TSs. The effect of the tested thiosemicarbazones was compared with the effect of mHtcum. Values are expressed as percentage of asci with respect to the number of total yeast cells; statistically significant differences from the control (DMSO) were indicated with different letters (*p* ≤ 0.01).

The anti-aflatoxigenic and anti-sclerotigenic activity of a cuminaldehyde TS derivative (mHtcum) was recently found to correlate with a gametogenesis reduction in yeast cells^35^. Thus, the correspondence between the biological effect of our compounds in *A. flavus* and asci production by *S. cerevisiae* diploid strain W303 was evaluated. As a general observation, all the tested compounds induced a decrease in the number of asci in treated cells (Fig. 5); however, significant differences among these molecules were documented: the less effective was BeTS (16% asci vs 24% of DMSO treatment), while CiTS proved to possess the highest potential (2.3% asci), showing an efficacy similar to mHtcum (2.9% asci), that was included in the assay as a reference. The two dimethylated derivatives shared the same level of gametogenesis inhibition (nearly 10% of asci).

### Regulation of gene expression

**Figure 6:**
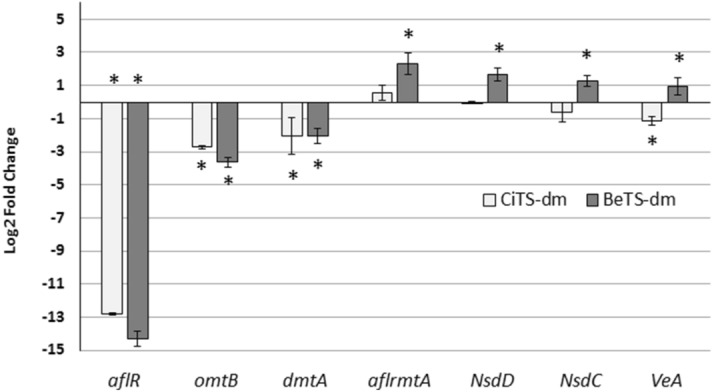
Regulation of gene expression in *A. flavus* treated with di-methylated TSs. Relative transcript levels of genes involved in fungal development (*VeA, NsdD, NsdC, aflrmtA and dmtA*) and AFs biosynthesis (*aflR* and *omtB*) evaluated in the same cultural conditions used for proteomic analysis (CCM medium amended with 50 μM compounds). Results are means of 3 replicates ± S.D. from 3 independent experiments. Asterisks mark values significantly different between treatments and control (0.5% DMSO-treated cultures) at *p* ≤ 0.05 (*t*-test).

An evaluation on the expression level of two genes belonging to the aflatoxigenic cluster (*aflR* and *OmtB*) and five genes linked to morphogenesis, development and secondary metabolism in *A. flavus* (*dmtA*, *aflrmtA*, *NsdD*, *NsdC*, and *VeA*) was then conducted in cultures treated with BeTS-dm or CiTS-dm at 50 µM concentration. Transcription level of each gene was assessed by RT-qPCR and reported as fold increase/decrease (Fig. 6). The expression of AF’s gene cluster regulator *aflR* was found dramatically repressed by the exposure of the fungus to both the dimethylated TSs (from 13 to 14 fold), as obtained, albeit to a lesser extent, for the structural gene *omtB* (threefold repression). Also the transcription of *dmtA* was slightly lowered by both the compounds (twofold). On the contrary, other genes tested showed a different behaviour molecule-dependent: *aflrmtA* (a gene encoding for an arginine methyltransferase protein, responsible for the post-translational methylation of arginine at the histones level and therefore involved in a range of important biological processes including signal transduction and epigenetic regulation^42^), *NsdD* and *NsdC* (positive regulators of asexual sclerotia, AFs biosynthesis and conidiophore development^43^) were not significantly affected by CiTS-dm treatment, as compared to the control cultures, but resulted slightly up-regulated in presence of BeTS-dm. *VeA*, a conserved regulatory gene unique to fungi and cooperating in sporulation processes and secondary metabolism management^44^, was also differentially affected in transcription: while slightly down-regulated by CiTS-dm, its expression level was not significantly altered by BeTS-dm treatment.

### Proteomic analysis

**Figure 7:**
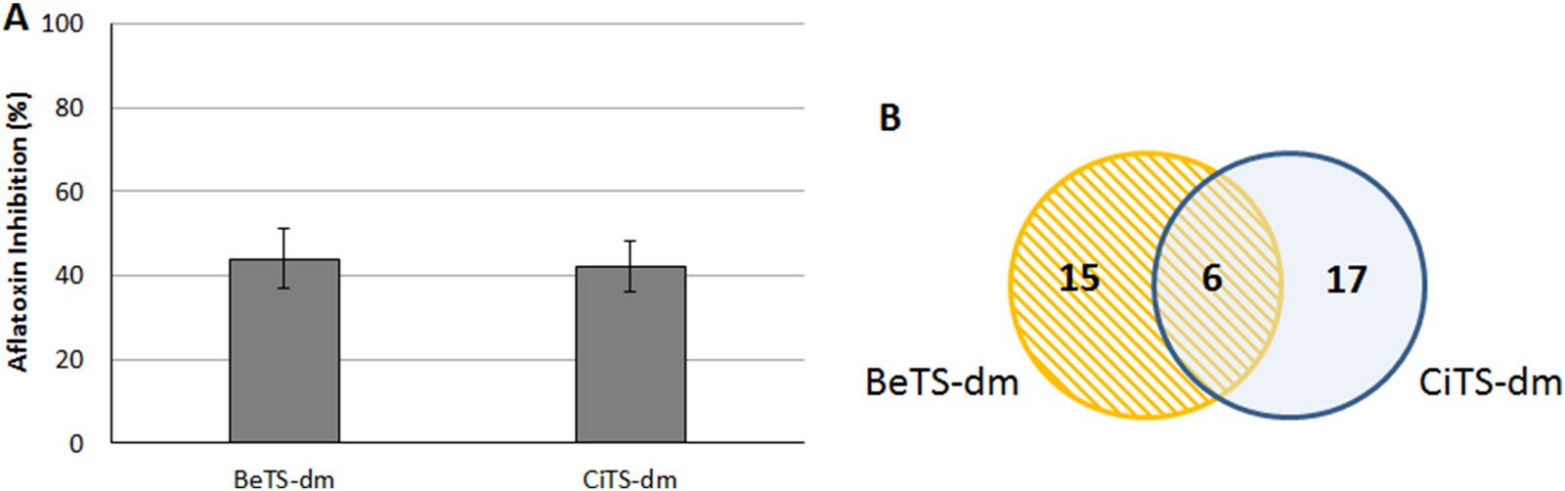
(**A**) Aflatoxin inhibition. BeTS-dm and CiTS-dm inhibitory effect on AF accumulation checked after 96 h exposure, before sampling mycelia for 2-DE analysis. (**B**) Differentially expressed proteins. Venn diagram indicating the overlap of differentially expressed spots between BeTS-dm (yellow circle) and CiTS-dm (blue circle) treatment, compared to the control (DMSO).

To deepen their differential activity, we investigated the changes in *A. flavus* proteome after CiTS-dm and BeTS-dm exposure. As recently reported for a set of TS-derived molecules^28^, a 2-DE analysis was conducted on total protein extracts from mycelia grown for 96 h in CCM medium amended with 50 µM TS (or 0.5% DMSO, as control; CNT). Parameters in PDQuest analysis software (*p* ≤ 0.01) were set in order to push the stringency to the maximum. AF accumulation was checked prior mycelium sampling (Fig. 7A). Comparison was conducted between BeTS-dm vs control (CNT), CiTS-dm *vs* control, and BeTS-dm *vs* CiTS-dm. A pH 3–10 coverage of the proteome was achieved: a total of 270 spots per gel were individuated; among them, 15 were found differentially expressed in CNT *vs* BeTS-dm, 17 in CNT *vs* CiTS-dm and 6 in BeTS-dm *vs* CiTS-dm (Fig. 7B). The low amount found in both TSs *vs* CNT comparison suggested that the molecular target/s of the molecules should be located down-stream the majority of primary metabolism processes essential for growth and development. At the same time, the smaller quantity of differential proteins individuated during BeTS-dm *vs* CiTS-dm comparison led us to speculate that the metabolic “distance” between the relevant targets could actually be relatively short. We focused on these 6 differential spots, proceeding with the MALDI-TOF/TOF identification. Results, reported in Table 1, showed that spots found to be differentially regulated by one treatment could be not differentially regulated by the other: for example, spot 5508, that corresponds to alcohol dehydrogenase, was found to be down-regulated in CiTS-dm treated mycelium, while no difference in protein abundance was observed in BeTS-dm treatment. Only spot 7710 (a Cu–Zn superoxide dismutase) showed to be differentially up-regulated and down-regulated in both treatments if compared to control.

**Table 1:** Differentially expressed proteins in BsTS-dm *vs* CiTS-dm comparison.

# Spot SSP	BeTS-dm vs CNT	CiTS-dm vs CNT	Accession number	Description matched protein	Score	Coverage (%)	MW (kDa)/pI
4208	↓	=	B8NHZ4	AflM/ ver-1/ dehydrogenase/ ketoreductase OS = *Aspergillus flavus*	99	8	28.107 / 6.31
4515	↑	=	A0A2P2HTF7	Acyl-coA-binding protein (ACBP) OS = *Aspergillus flavus*	150	18	15.569/5.61
5311	=	↑	A0A0F0IEE3	Glyceraldehyde-3-phosphate dehydrogenase OS = *Aspergillus flavus*	72	3	40.707/8.94
5508	=	↓	A0A2P2GYJ6	Alcohol dehydrogenase OS = *Aspergillus flavus*	265	15	37.402/6.34
7710	↑	↓	A0A2P2HKD7	Superoxide dismutase [Cu–Zn] OS = *Aspergillus flavus*	94	14	16.913/6.03
6609	↓	=	A0A2P2HBZ5	WD domain G-beta repeat OS = *Aspergillus flavus*	167	12	35.395/6.21

Variation in spot abundance between BeTS-dm and CiTS-dm treatment (50 μM), and control (0.5% DMSO) is indicated with a upward (↑) or downward (↓) pointing arrow for up-regulated and down-regulated peptides respectively. Fold changes were considered statistically significant when exceeded both a fold of variation > 2.0 (*p* ≤ 0.01).

## Discussion

Many strategies have been devised to reduce AFs diffusion, but the use of chemical agents is still the most effective in controlling post-harvest contamination. However, due to some drawbacks in their application (such as toxicity, residues in food chain and occurrence of resistance phenomena), the importance of discovering new efficient and safe substances for preventing and controlling *A. flavus’*s growth and AFs production is paramount. Countless are the studies highlighting potent anti-fungal activity of natural compounds: prominently, essential oils such as carvacrol, eugenol, citral and cinnamaldehyde, were found to be highly promising^45–48^.

The use of natural products as scaffolds could therefore be a privileged starting point to obtain selective and highly potent antifungals. Natural products are in fact in most cases secondary metabolites produced by organisms to protect themselves from natural antagonists, predators or pathogens, as well as from the environment. Natural product–based drug discovery takes therefore advantage of a scaffold characterized by being already biologically active and, at least in principle, it should be possible by specific modifications in the structure to enhance its pre-existing biological activity, to obtain information about the molecular target or to add properties that improve drug delivery criteria. Aiming in particular at the first two issues, purposive modifications have been applied to benzaldehyde and cinnamaldehyde in order to raise their selectivity and their activity power. The addition of thiosemicarbazide to natural compound derivatives was successfully used to obtain TSs with enhanced antifungal and anti-aflatoxigenic properties^25,26,28–30^. Albeit to a different extent, a significant increase of toxin containment was obtained with the TS derivatives of both aldehydes BeTS, and CiTS (Fig. 3), as for the biomass reduction, and, in the case of Cinn, also for the early growth of mycelium. Even if the general mechanism of action of TSs on fungi is still debated, studies revealed that they seem to be able to induce changes in cell membrane biosynthesis/composition. In particular, it was reported how some TSs can influence the synthesis and regulation of ergosterol^49,50^, that is strictly connected with the metabolism of lipids and cell membrane synthesis, and hence essential for fungal hyphae branching and elongation. However, lipid metabolism is also connected with developmental processes belonging to secondary metabolism: AF biosynthetic pathway and sclerotia biogenesis are both dependent, and regulated, by acetyl-CoA intracellular availability^51–53^. Additionally, these processes have long been demonstrated to share various metabolic knots, even if the majority of them are still to be unravelled^53^. This was coherent with our observation that the increase in anti-aflatoxigenic activity of TSs was accompanied by a huge inhibitory effect on sclerotia production in most cases (Fig. 4).

Various findings ascribed the antitoxigenic activity of many compounds to their ability in modifying the intracellular redox balance in fungal hyphae, acting as ROS scavenging agents^34,40,52,54,55^; on the other hand, it has been often reported that the scavenging potential of TSs estimated with in vitro chemical assays could be not translated in a coherent in vivo effect on secondary metabolism^28^. Here, a consistent correlation between the in vitro anti-oxidant activity and the interference with AFs accumulation and sclerotia biogenesis was observed for Cinn TSs derivatives (CiTS and CiTS-dm) whereas, notably, the remarkable scavenging potential of BeTS-dm was coupled to AFs inhibition but not to any sclerotia reduction (Fig. 4A).

The ability to induce mitochondria impairment is one of the latest mechanisms proposed to explain the biological activities of some TSs^35^. In fact, the inhibition of the electron flow through the mitochondrial respiratory chain proved to exert, at various levels, the disruption of cellular respiration, leading to a progressive blockage of specific developmental processes in fungi^56–58^. Recently, we described cytochrome bc_1_ complex (complex III) as the putative target of 3-isopropylbenzaldehyde TS (mHtcum), a cuminaldehyde derivative highly effective in lowering AF accumulation and sclerotia production^35^. By using the model system *Saccharomyces cerevisiae* it was observed that mHtcum interferes with gametogenesis, a mitochondrial respiratory-linked process for which regulation the carbon/energy status plays a critical role in yeast. When compared with mHtcum, the effects of BeTS, CiTS and the dimethylated derivatives showed interesting dissimilarities: the lower efficiency of CiTS-dm with respect to its parent CiTS traced what observed in early mycelium development and biomass production assays (Fig. 3), while, on the contrary, BeTS-dm resulted more effective than BeTS in reducing yeast gametogenesis as for AFs containment. Apparently, methylation of the NH_2_ moiety, that was thought in order to modulate the lipophilicity and hydrogen-bonding capabilities of the parent TSs, seems to worsen the effect of CiTS on mitochondrial functionality but to improve the activity of BeTS, rendering the activity of the two dimethylated derivatives very similar.

CiTS-dm and BeTS-dm were found to possess a similar behaviour when their effect was compared at gene transcription level, as evaluated in treated *A. flavus* cultures exposed to 50 µM solutions of the two compounds. The whole signalling network for cellular processes regulating the AF metabolism is still to be unravelled, but several components of these networks have been characterized: AFs production in *A. flavus* is managed by a 72 kb gene cluster, which products establish an enzymatic cascade involving at least 21 steps, regulated by aflR and aflS transcriptional factors^59,60^; a significant decrease in the transcription level of these two genes has been demonstrated to correlate with the suppression of AF accumulation, through the down-regulation of different structural genes in the AF cluster^61^. Hence, it was not surprising that, consistently with the AF accumulation reduction assessed (Fig. 3), a dramatic decrease of *aflR* expression was recorded in both CiTS-dm and BeTS-dm treatments (Fig. 6), and, as a consequence, also the expression of the aflatoxigenic cluster structural gene *OmtB* resulted impaired: the decline in AF biosynthesis is clearly on account of the down-expression of at least these two key genes belonging to the AF cluster. These observations partially differ with the results achieved during previous studies on cinnamaldehyde, whose inhibitory effects on fungal development and mycotoxin production are well documented, that reported a significant down-regulation of all the structural genes of the aflatoxigenic cluster alongside a slight up-regulation of the two regulators aflR and aflS^46,47,62^. On the other hand, our results showed a decrease in expression level for *dmtA*, a putative C-5 cytosine methyltransferase essential for AF production and sclerotia biogenesis in *A. flavus*, supporting the hypothesis of a role played by DNA methylation (and/or DNA methyltransferases) in the fungal secondary metabolism^16^. However, unlike what has been noticed in CiTS-dm treated mycelia, that exhibited an 80% reduction in sclerotia production, the sclerotial biomass in cultures exposed to BeTS-dm did not differ from the control as it should have been expected (Fig. 4). In this sense, the difference between the two dimethylated TSs regarding *aflrmtA*, *NsdD* and *NsdC* genes expression might partially explain these results. The transcription factors NsdC and NsdD, both required for the production of sclerotia^43^ and supposed to display their role upstream of AF biosynthetic and sclerotia developmental pathways, are known to be positively regulated by *aflrmtA* gene. Therefore, the increase of *aflrmtA* expression level observed in BeTS-dm treated cultures seems to be consistent with both the slight up-regulation of *NsdD* and *NsdC* genes and the absence of any inhibitory effect on sclerotia production.

A different effect of the two dimethylated TSs was evidenced also in the case of *VeA*, a global regulatory gene governing development and secondary metabolism in numerous fungal species including *A. flavus*. Together with other transcription factors, *VeA* has been demonstrated to mediate oxidative stress-responsive signalling that is involved in AF biosynthesis^13,40,63^, and its presence is considered critical for sclerotia biogenesis^64^. Hence, the slight down-regulation of expression level fits with down-regulation of AF production and AF related genes, and sclerotia deficiency in CiTS-dm treated cultures. On the contrary, BeTS-dm activity repressed AFs genes when up-regulated *VeA* and did not resulted in sclerotia development impairment, suggesting that at least two different cellular targets might exist for this compound. The finding that the only differential spot found to be up-regulated by BeTS-dm and down-regulated by CiTS-dm treatment corresponds to a Cu/Zn-dependent superoxide dismutase, additionally supports this hypothesis: in fact, the deletion of the *VeA* gene resulted, in *A. flavus* ΔVeA mutants, in a higher SOD activity that concurs with a decrease of oxidative stress to such a low level that both sclerotia and aflatoxin B_1_ production are inhibited^65,66^.

The analysis of proteome alteration also showed a different decrease of aflM (ver-1) protein in *A. flavus* mycelium treated with BeTS-dm: the *aflM* gene is known to be one of the four genes involved in the conversion of versicolorin A into demethylsterigmatocystin, one of the last enzymatic steps of AFB_1_ biosynthesis, and its expression was demonstrated to depend on the regulator *aflR* gene activity^67^. However, under certain AF inhibitory conditions, a marked decrease in cluster gene expression and AF production was observed with variable changes in aflR expression level^68^, suggesting that modified ratios of available AflR/AflS proteins could lead to the formation of an insufficient number of complexes; as a consequence, all the AflR-binding sites on the AF cluster may not be reached, and the subsequent cluster transcription is not complete. Since genes encoding enzymes involved in the final stages of the AFB_1_ enzymatic cascade seem to be more affected than those involved in the initial steps, the hypothesis is that the limited number of AflR/AflS complexes available might have been rapidly used at the beginning of AF biosynthesis, and were no longer available to properly activate the last cluster genes, as *aflM* ^67^. In this scenario, the inhibition of AF accumulation induced by BeTS-dm seems to be more associated to a late-interference on AF pathway with respect to CiTS-dm, even if the two compounds determined a similar decrease in *aflR* gene expression. Differential abundance of alcohol dehydrogenase (ADH) support this hypothesis: in fact ADH, an enzyme involved in carbon/energy metabolism, resulted down-regulated by CiTS-dm exposure respect to control, while no difference was found after BeTS-dm treatment; similar results were already reported for mHtcum, which was effective in lowering both AF and sclerotia biosynthesis^28^, suggesting for the dimethylated form of CiTS at least one possible target up-stream AF and sclerotia-forming processes.

On the other hand, the Acyl-CoA-binding protein (ACBP) up-regulation by BeTS-dm appeared inconsistent with the previously discussed results: β-oxidation of fatty acids in peroxisome and mitochondria is a major contributor to acetyl-CoA, the fundamental structure element of all fungal polyketides such as AFs, and the competition for acetyl-CoA between lipid synthesis and polyketides formation has been proven by the observation that several genes involved in fatty acids β-oxidation were down-regulated by chemicals or conditions inhibiting AF biosynthesis^62,69,70^. It was also shown that ACBP, in contrast with fatty acid binding protein (FABP), stimulates the synthesis of long-chain acyl-CoA esters by mitochondria, effectively opposing the product feedback inhibition of the long-chain acyl-CoA synthetase by sequestration of the synthesized acyl-CoA esters; therefore, the combined above results suggested us that the AFs blockage induced by BeTS-dm activity might in turn reflect on the intracellular pool of acyl-CoA, interfering with the mitochondrion-peroxisome interplay.

## Conclusions

**Figure 8:**
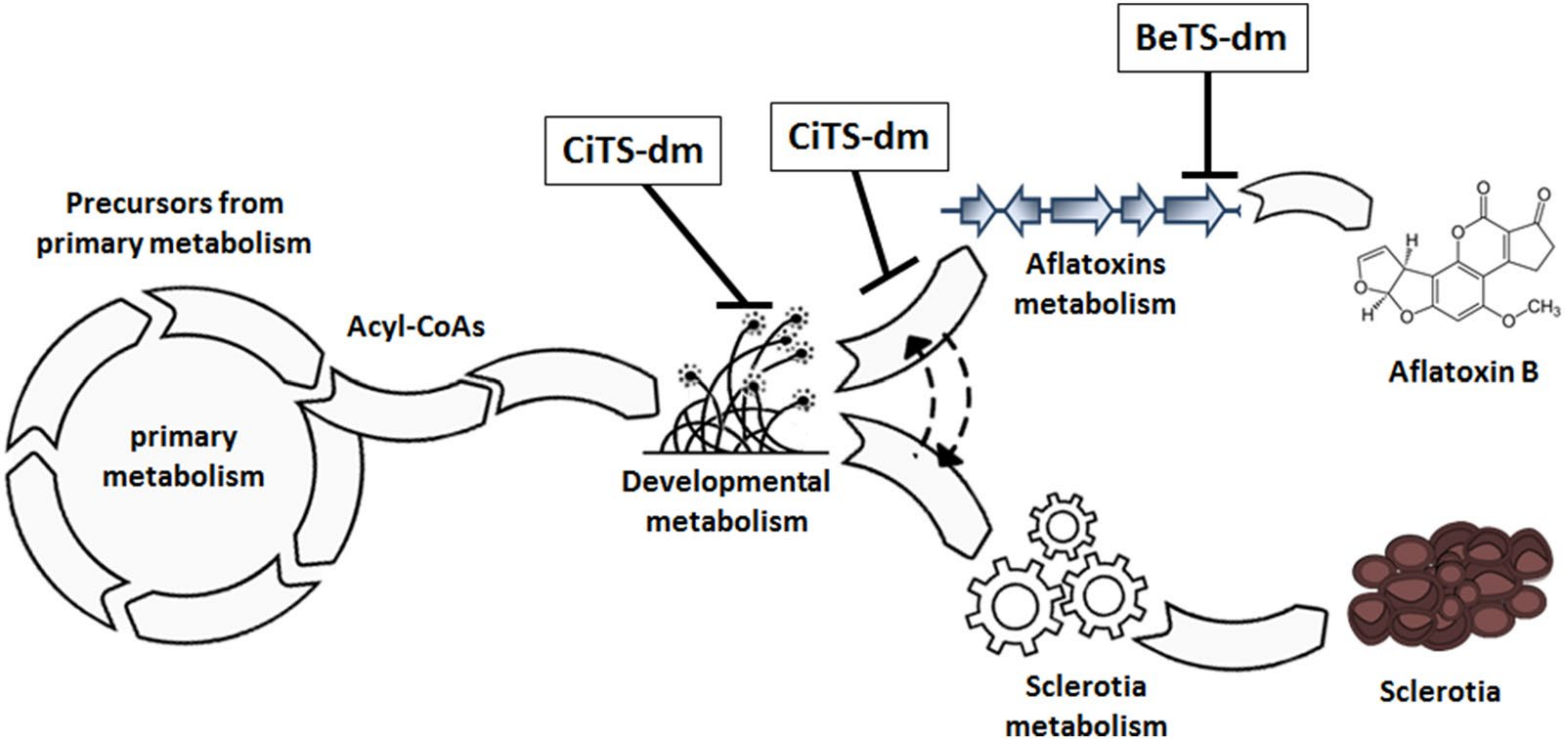
Hypothetical targets positioning of BeTS-dm and CiTS-dm along *A. flavus* secondary metabolism. Being ineffective on sclerotia biogenesis while highly inhibitory on AF accumulation, BeTS is suggested to intervene down-stream the bifurcation of secondary metabolism that divide AFs biosynthetic pathway from sclerotia developmental biogenesis. On the contrary, due to the containment effect of CiTS-dm on sclerotia and AF, its target could be expected enough up-stream the bifurcation to interfere with their production, or immediately after, in correspondence of a metabolic knot shared by (and controlling) both processes in *A. flavus*. Scheme created with Adobe Illustrator and Adobe Photoshop CS6.0 (Adobe Inc., San Jose, CA, USA).

On the basis of the reported results, it has been here evidenced that the tested TSs possess biological activities, different in intensity and features, which depend on the chemical scaffold and on the structural modifications introduced. Our findings demonstrate that the dimethylated cinnamaldehyde and benzaldehyde TS derivatives BeTS-dm and CiTS-dm are highly effective compounds, able to differentially inhibit AFs production and/or sclerotia biogenesis in *A. flavus* cultures. CiTS-dm, in particular, has proven to be an extremely selective anti-aflatoxigenic and anti-sclerotigen molecule, becoming significantly interesting for applications aimed to avoid mere fungicidal, nonspecific agents. A deeper investigation into the properties of these compounds revealed the existence of different cell targets, located along the fungal developmental and secondary metabolism. In the proposed model depicted in Fig. 8, BeTS-dm was suggested to act directly on the AF pathway, downstream the bifurcation between AFs and sclerotia metabolism, accordingly to an inhibitory effect on toxin accumulation unrelated to sclerotia suppression. On the other hand, CiTS-dm seems to interfere upstream of the same bifurcation, intervening in the blockage of both processes. However, a different hypothesis, which justifies the anti-aflatoxigenic and anti-sclerotigen activity of CiTS-dm, could be inferred: in fact, recent is the discovery that “secondary” ROS produced during AFs biosynthesis contribute to the increase of primary ROS, thus driving the redox balance of the cell toward an oxidative state, a condition considered critical for triggering the sclerotia biogenesis^41^. In this scenery, if the CiTS-dm target (or one of them) is located on the AF pathway but upstream of the BeTS-dm target, the early shutdown of the subsequent enzymatic reactions might cause a low amount of secondary ROS, lower than those allowed by the activity of BeTS-dm, hence clarifying both its inhibitory effects on *A. flavus* secondary metabolic processes.

## Supplementary information

**Figure S1.** Effect of the tested compounds on *A. flavus*. The inhibitory activity of the molecules on early development (**A**), biomass production (**B**) and aflatoxin accumulation (**C**) was evaluated. Increasing concentrations were tested (25—50—100 µM) and results were expressed as percentage with respect to control (0.25 – 0.5 – 1% DMSO respectively); *p* value ≤ 0.01.

Supplementary file2
