# Supplementary figures and images for: Sisters in structure but different in character, some benzaldehyde and cinnamaldehyde derivatives differentially tune Aspergillus flavus secondary metabolism
Source: Sci Rep. 2020 Oct 19;10:17686. doi: 10.1038/s41598-020-74574-z (PMC7572373; doi:10.1038/s41598-020-74574-z)

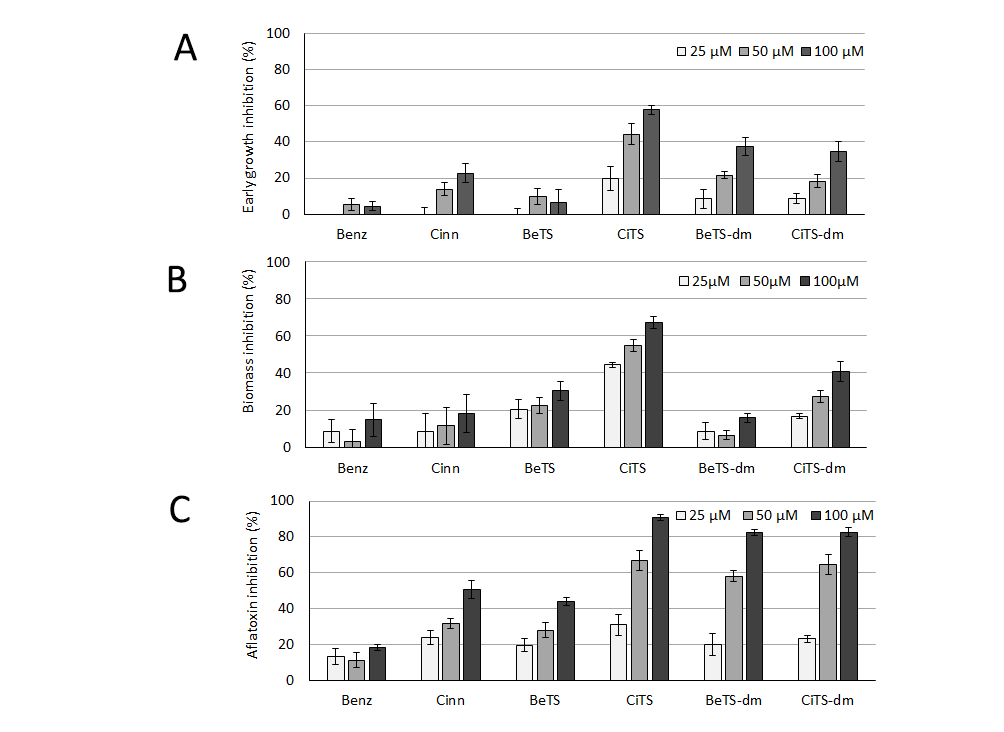

Supplement: Supplementary file 1 — Figure S1. Effect of the tested compounds on A. flavus. The inhibitory activity of the molecules on early development (A), biomass production (B) and aflatoxin accumulation (C) was evaluated. Increasing concentrations were tested (25—50—100 µM) and results were expressed as percentage with respect to control (0.25 – 0.5 – 1% DMSO respectively); p value ≤ 0.01. [file 41598_2020_74574_MOESM1_ESM.tif]
