# Supplementary material for: Sisters in structure but different in character, some benzaldehyde and cinnamaldehyde derivatives differentially tune Aspergillus flavus secondary metabolism
Source: Sci Rep. 2020 Oct 19;10:17686. doi: 10.1038/s41598-020-74574-z (PMC7572373; doi:10.1038/s41598-020-74574-z)
Supplement: Supplementary file 2 — Supplementary file2 [file 41598_2020_74574_MOESM2_ESM.docx]

Table 1S

| Primer | Sequence (5’3’) | Gene target |
| --- | --- | --- |
| tubFw-RT ^28^ | TACCATGGACGCCGTCCG | *tub1* |
| tubRev-RT ^28^ | GACGGACAACATCGACAAC |  |
| AflRFw-RT ^35^ | CTATTGAAGCCCTTGACAC | *aflR* |
| AflRRev-RT ^35^ | GAGTCTCTTACCTACACCC |  |
| OmtBFw-RT ^28^ | ACGGATCACTTAGCCAGCAC | *omtB* |
| OmtBRev-RT ^28^ | ACGGTGCTTTTGGGACGTTG |  |
| dmtAFw-RT ^63^ | AAGAGGCGGCGACAGTATA | *dmtA* |
| dmtARev-RT ^63^ | GATAGGTAGAGGTTGCGTGTTC |  |
| aflrmtAFw-RT ^42^ | CAGCATGTATGTGGTCAGAG | *aflrmtA* |
| aflrmtARev-RT ^42^ | GTCCGACAGGCCGTTGCAAG |  |
| NsdDFw-RT ^28^ | GTCACGACTCAAGATTCGC | *NsdD* |
| NsdDRev-RT ^28^ | ATGCTGCCCGCAGAAGTAG |  |
| NsdCFw-RT ^28^ | CGAGAAGCAACAACTAAATACAAT | *NsdC* |
| NsdCRev-RT ^28^ | TTGCAACTGTGGAGGTATTC |  |
| VeAFw-RT ^28^ | CGAGACGGAAGCCTCCGT | *VeA* |
| VeARev-RT ^28^ | TGGAGGATCGACTGGACGA |  |

**Table 1S. List of oligonucleotides used for qRT-PCR analysis.** Oligos were previously described by different authors. Superscript numbers refer to the relevant literature.
